# Morganella morganii Detected During Transfemoral Amputation in a Diabetic Foot With Soft Tissue Necrosis

**DOI:** 10.7759/cureus.117901

**Published:** 2026-10-14

**Authors:** Arın Celayir, Ibrahim Dikçi, Bedri Karaismailoglu

**Affiliations:** 1 Department of Orthopaedics and Traumatology, Çukurca State Hospital, Hakkari, TUR; 2 Department of Orthopaedics and Traumatology, Istanbul University-Cerrahpasa, Cerrahpasa Medical School, Istanbul, TUR

**Keywords:** diabetic foot, morganella morganii, musculoskeletal infection, necrotizing soft tissue infection, transfemoral amputation

## Abstract

This case report describes a 63-year-old man with diabetes mellitus and peripheral arterial disease who was referred to our clinic with circulatory insufficiency and a foul-smelling necrotizing wound of the right lower extremity. The patient had previously undergone bilateral balloon angioplasty, which restored distal pulses; however, progressive soft tissue deterioration led to extensive purulent ulcers along the anteromedial and posterior aspects of the lower leg. Imaging demonstrated subcutaneous gas in the posterior soft tissues, and transfemoral amputation was performed.

During surgical dissection, purulent discharge was observed along the anteromedial aspect of the femur, and intraoperative samples were obtained for microbiological analysis. Cultures yielded *Enterobacter species* and *Morganella morganii*, the latter being an opportunistic Gram-negative bacillus that is rarely implicated in musculoskeletal infections.

This case is notable for the uncommon identification of *Morganella morganii* in a deep musculoskeletal infection and emphasizes the importance of broad microbiological consideration in necrotizing soft tissue infections, particularly in patients with diabetes or other predisposing conditions. Timely surgical intervention, adequate source control, and culture-directed antimicrobial therapy remain essential in such complex clinical scenarios.

## Introduction

Lower extremity wounds are a major complication of diabetes mellitus and are often aggravated by peripheral neuropathy, impaired immune response, and vascular dysfunction. Although macrovascular circulation may be restored through endovascular or surgical intervention, diabetic foot ulcers and associated soft-tissue damage can continue to progress because of persistent local tissue compromise [1-3]. These wounds remain a major cause of non-traumatic lower limb amputation.

Diabetic foot infections are frequently polymicrobial and may involve aerobic and anaerobic organisms. *Staphylococcus aureus*, *Escherichia coli*, and *Pseudomonas aeruginosa* are commonly encountered, whereas less common pathogens may be isolated in chronic or necrotizing wounds. *Morganella morganii* is an opportunistic, Gram-negative, facultatively anaerobic bacillus that is part of the normal intestinal flora and has been associated with urinary tract, bloodstream, and postoperative infections [4,5]. Its involvement in deep soft tissue and musculoskeletal infections is uncommon [5].

The identification of *Morganella morganii* in a lower extremity wound requiring major surgical debridement or amputation is clinically noteworthy because the organism may represent either colonization or true invasive infection. This report presents a case in which *Morganella morganii* was detected during transfemoral amputation for an advanced diabetic wound with extensive soft tissue necrosis after lower extremity revascularization.

## Case presentation

A 63-year-old man was referred to our clinic from an external center because of circulatory insufficiency of the right foot. His medical history included diabetes mellitus and peripheral arterial disease. He was not receiving regular antimicrobial therapy before admission. Laboratory testing showed a hemoglobin level of 10.3 g/dL and an elevated C-reactive protein level of 96 mg/L, consistent with systemic inflammation.

On physical examination, purulent discharge extended from the anterior and posterolateral aspects of the right lower leg to its midportion. Bilateral sensory deficits were present in the lower extremities (Figure 1).

**Figure 1:**
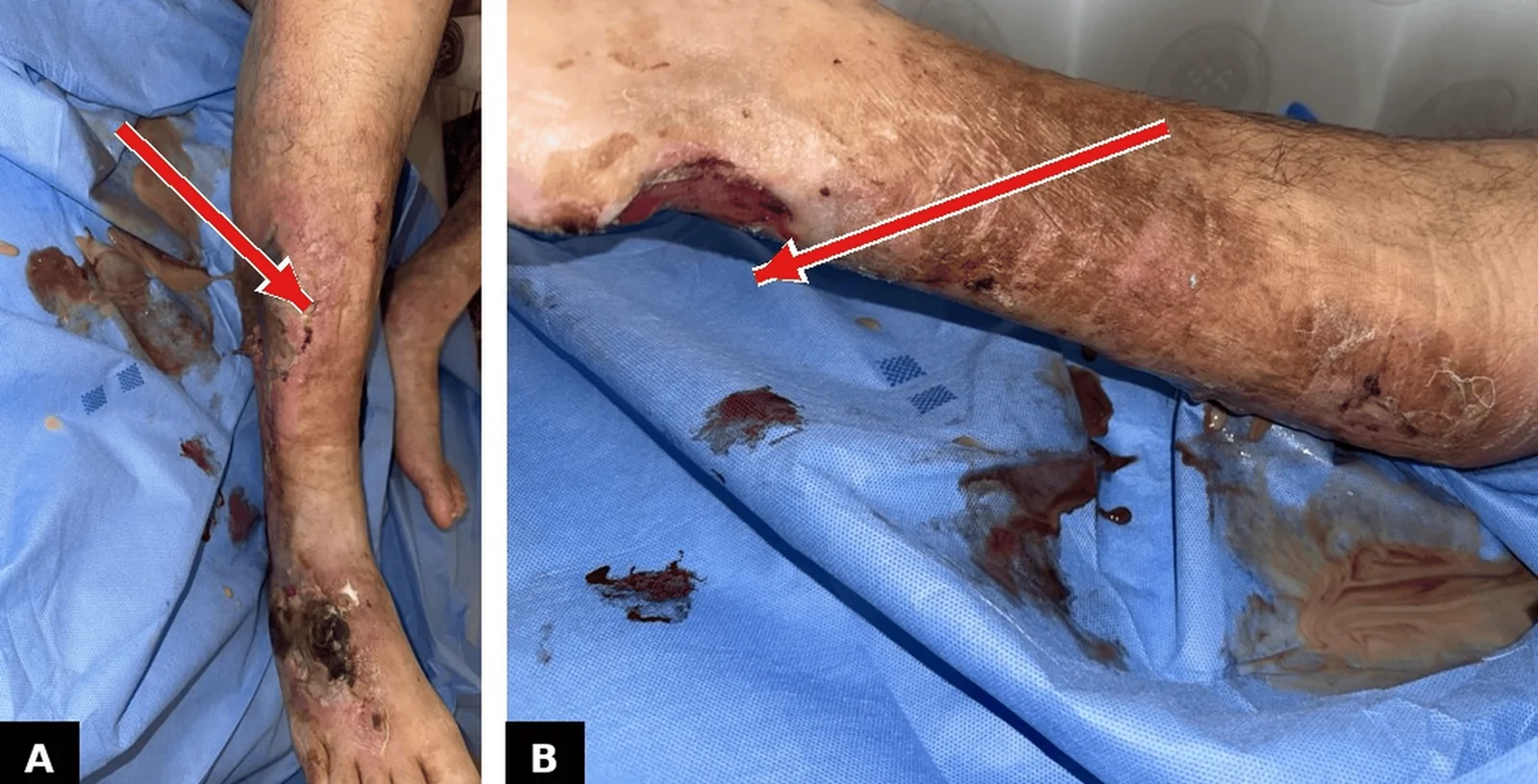
Clinical appearance of the right lower extremity Extensive necrotic, infected, and discharging wounds are visible on the cruris. (A) Anterolateral view showing extensive soft-tissue necrosis and purulent wound areas. (B) Posterior view demonstrating the posterior crural wound and surrounding compromised soft tissue. Red arrows indicate the principal wound areas.

The patient was admitted to the orthopedic ward, and preoperative preparations were initiated. Because of the elevated inflammatory markers and extensive soft-tissue infection, the Infectious Diseases team was consulted. Empirical antimicrobial therapy was initiated with piperacillin-tazobactam 4.5 g intravenously 3 times daily and teicoplanin (800 mg loading dose followed by 400 mg every 12 hours).

The ulcer on the right foot had developed approximately four months earlier. The patient had undergone bilateral lower extremity balloon angioplasty at an external facility, resulting in restoration of previously compromised arterial flow and palpable popliteal pulses. Despite improved macrovascular circulation, progressive soft tissue necrosis and infection necessitated transfemoral amputation.

Preoperative radiographs demonstrated gas densities within the posterior soft tissues of the lower leg (Figure 2).

**Figure 2:**
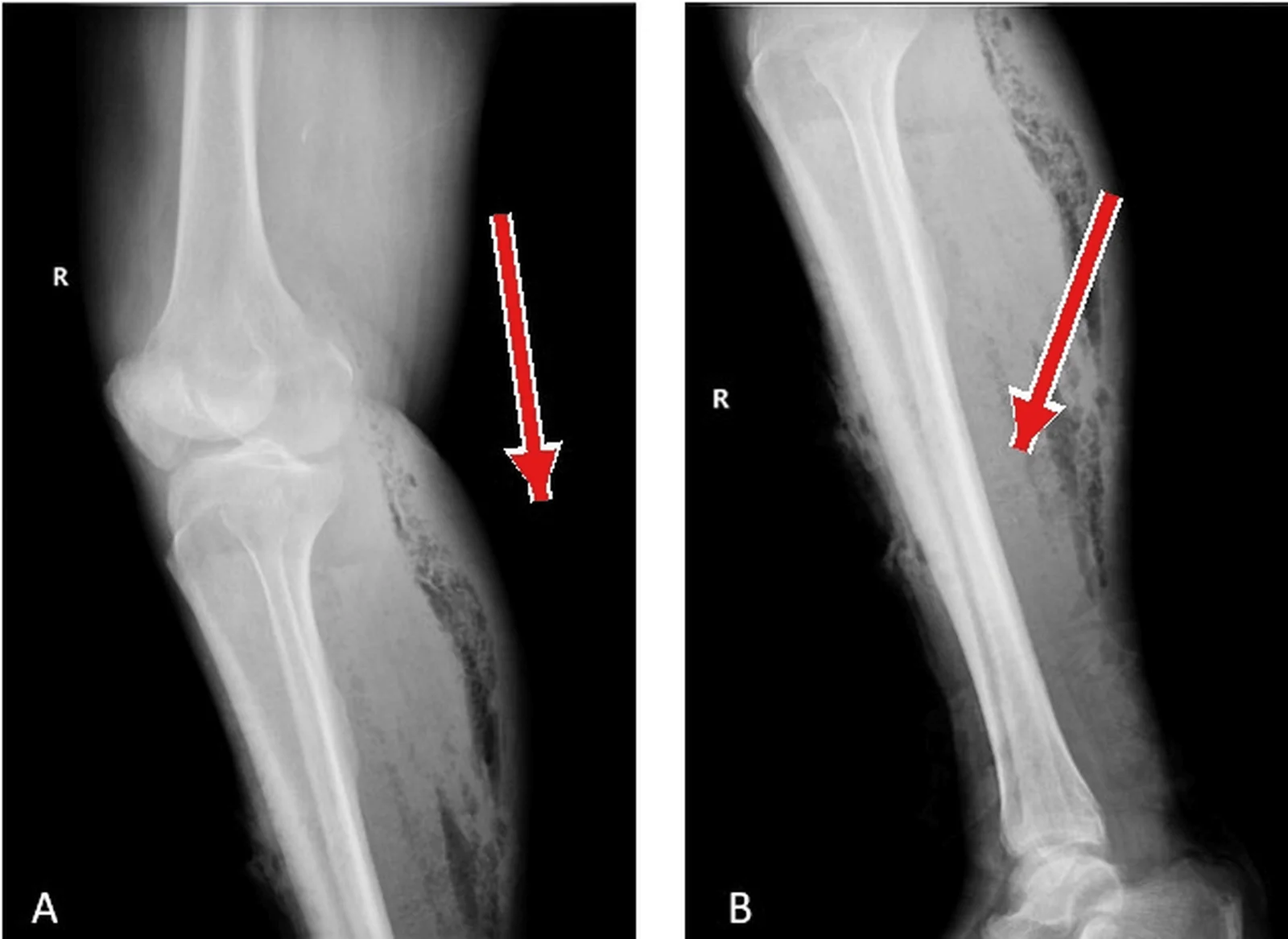
Preoperative anteroposterior and lateral radiographs of the right cruris (A) Anteroposterior view. (B) Lateral view. Red arrows indicate the soft-tissue gas. Extensive gas densities are visible within the posterior soft tissues of the cruris.

During surgery, purulent discharge was encountered along the anteromedial aspect of the femur during soft tissue dissection before the femoral osteotomy. Samples were obtained for microbiological analysis. The femoral osteotomy was then completed, and the surgical wound was closed.

The patient was transferred to the intensive care unit postoperatively. No unexpected abnormality was identified on postoperative imaging (Figure 3).

**Figure 3:**
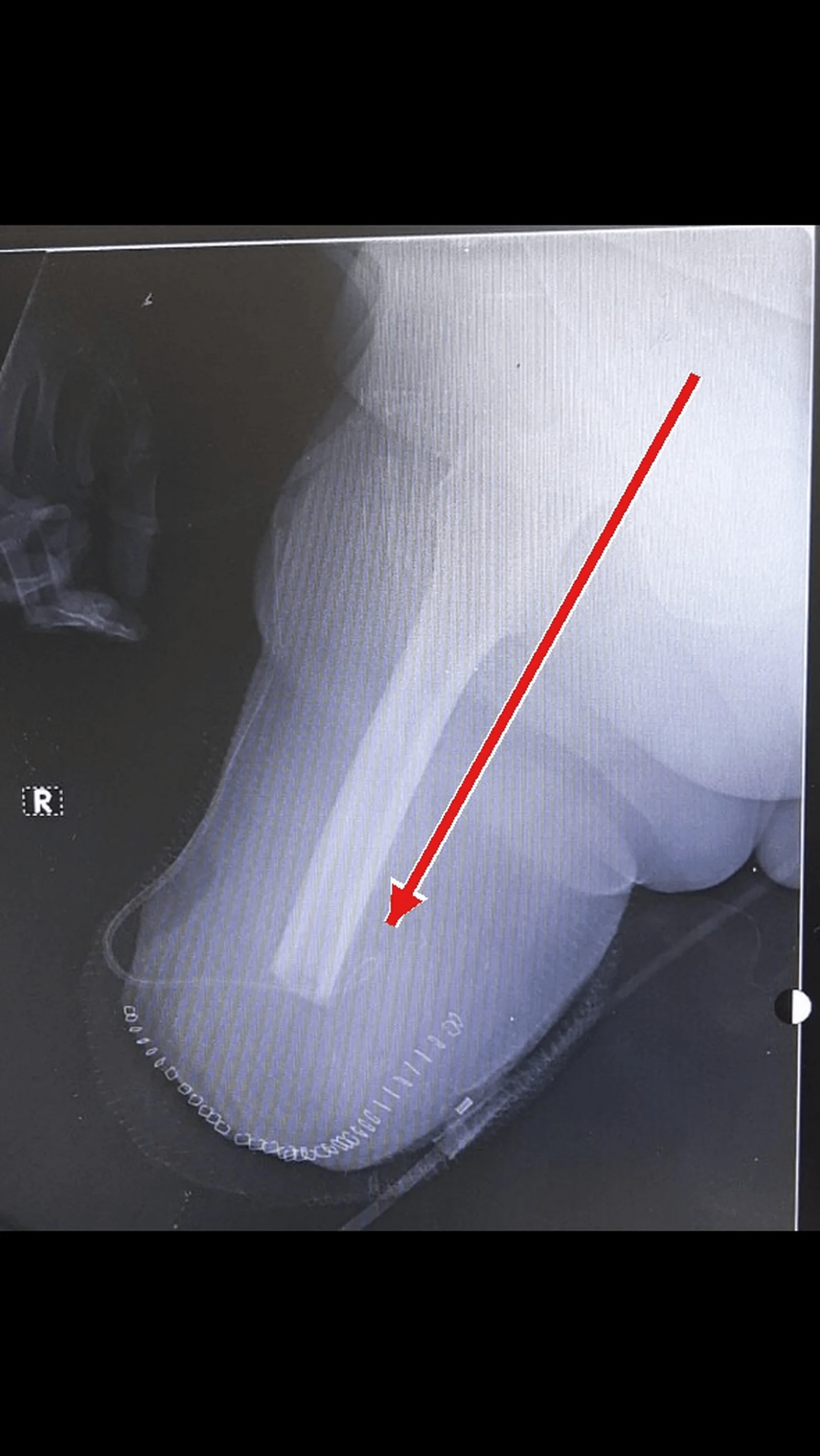
Postoperative radiograph of the right femur following transfemoral amputation The red arrow indicates the amputation level and postoperative femoral stump.

During inpatient follow-up, intraoperative cultures yielded *Enterobacter *species and *Morganella morganii*. The Infectious Diseases team was consulted again, and antimicrobial therapy was adjusted according to the microbiological findings and susceptibility results. Given the potential for inducible AmpC beta-lactamase production in *Morganella morganii*, antimicrobial selection was guided by the susceptibility profile rather than by empirical coverage alone. The amputation stump was monitored closely with serial wound examinations, assessment for erythema, drainage, dehiscence, and recurrent necrosis, as well as follow-up of systemic inflammatory parameters. The postoperative wound remained clinically stable without evidence of recurrent purulent drainage, progressive soft-tissue infection, or wound dehiscence during the inpatient period. Postoperative radiographs demonstrated no acute abnormality. The patient continued to receive multidisciplinary care involving the Orthopaedics and Infectious Diseases teams, with culture-directed antimicrobial therapy and routine stump care.

## Discussion

Diabetic foot ulcers are among the most challenging complications of diabetes mellitus and may lead to progressive soft tissue destruction, infection, and eventual amputation. Although macrovascular circulation can often be improved through endovascular or surgical procedures, revascularization does not always prevent tissue loss when neuropathy, impaired host defenses, chronic ulceration, and established infection persist [1-3]. In the present case, extensive cutaneous and subcutaneous tissue necrosis progressed despite technically successful revascularization and palpable pulses, illustrating the multifactorial nature of limb-threatening diabetic wounds.

Infectious complications play a central role in the progression of diabetic foot wounds. These infections are often polymicrobial and may include aerobic Gram-positive cocci, Gram-negative bacilli, and anaerobic organisms. The detection of less common organisms, such as *Morganella morganii*, may therefore have important implications for antimicrobial selection and source control.

*Morganella morganii* is a facultatively anaerobic, motile Gram-negative rod and an opportunistic component of the normal gastrointestinal flora. It is most frequently associated with urinary tract, bloodstream, hepatobiliary, and postoperative infections, particularly in hospitalized or immunocompromised patients [4,5]. Musculoskeletal involvement is uncommon, and published reports are limited compared with infections at other sites [5].

In this case, *Morganella morganii *was isolated from purulent material encountered intraoperatively during transfemoral amputation. The purulence was located along the anteromedial aspect of the femoral shaft, suggesting deep soft tissue involvement and raising concern for early osseous extension. Although isolation from a chronic wound can sometimes represent colonization, the concurrent necrosis, radiographic gas, purulent drainage, and elevated inflammatory markers supported a clinically significant infectious process.

The literature on *Morganella morganii *in musculoskeletal and necrotizing soft tissue infections remains limited. Kakurai et al. described a necrotizing soft tissue infection caused by *Morganella morganii* and emphasized the organism’s potential for aggressive local invasion and rapid tissue destruction, particularly in susceptible hosts [6]. Their report also highlighted the importance of early recognition, surgical source control, and microbiologically guided antimicrobial therapy.

Similarly, Koyuncu and Ozan reported *Morganella morganii* osteomyelitis complicated by septic arthritis of the knee, one of the uncommon orthopedic infections attributed to this organism [7]. *Morganella morganii* has also been reported as an unusual pathogen in infected joint effusions [8]. These reports support the view that *Morganella morganii* should not automatically be dismissed as a contaminant when isolated from deep musculoskeletal specimens, particularly in patients with chronic wounds, diabetes, or prior healthcare exposure.

Antimicrobial resistance is an important consideration in *Morganella morganii* infection. The organism can produce inducible AmpC beta-lactamases and has the potential to develop multidrug resistance, complicating empirical treatment choices [5]. Accordingly, deep intraoperative sampling, organism identification, and antimicrobial susceptibility testing are important in advanced diabetic limb infections, particularly when uncommon Gram-negative organisms are recovered.

This case adds to the limited literature describing *Morganella morganii* as a potential causative or co-contributive organism in deep musculoskeletal infection. Awareness of uncommon pathogens is important in complex diabetic wounds because delayed recognition may postpone appropriate culture-directed therapy. In addition to antimicrobial treatment, definitive surgical source control remains central when extensive necrosis and deep infection are present.

## Conclusions

*Morganella morganii* is an uncommon but clinically relevant pathogen in deep musculoskeletal and diabetic limb infections. When it is isolated from deep operative specimens in the setting of purulence, tissue necrosis, radiographic gas, and systemic inflammation, its potential pathogenic role should be considered rather than assuming contamination. Comprehensive intraoperative sampling, culture-directed antimicrobial therapy, and timely surgical source control are important for managing these complex infections.
